# The Electrospray (ESI) and Flowing Atmosphere-Pressure Afterglow (FAPA) Mass Spectrometry Studies of Nitrophenols (Plant Growth Stimulants) Removed Using Strong Base-Functionalized Materials

**DOI:** 10.3390/ma14216388

**Published:** 2021-10-25

**Authors:** Mateusz Pawlaczyk, Michał Cegłowski, Rafał Frański, Joanna Kurczewska, Grzegorz Schroeder

**Affiliations:** Faculty of Chemistry, Adam Mickiewicz University in Poznań, Uniwersytetu Poznańskiego 8, 61-614 Poznań, Poland; m.ceglow@amu.edu.pl (M.C.); franski@amu.edu.pl (R.F.); asiaw@amu.edu.pl (J.K.); schroeder@amu.edu.pl (G.S.)

**Keywords:** nitrophenols, functional silica, functional polymers, mass spectrometry, plant biostimulator

## Abstract

The functional silica-based materials functionalized with a strong nitrogen base TBD (SiO_2_-TBD) deposited via a linker or with a basic poly(amidoamine) dendrimer containing multiple terminal amine groups -NH_2_ (SiO_2_-EDA) and functional polymers containing a strong phosphazene base (Polymer-Phosphazene) or another basic poly(amidoamine) dendrimer (PMVEAMA-PAMAM) were tested as sorbents dedicated to a mixture of nitrophenols (*p*-nitrophenol and 2-methoxy-5-nitrophenol), which are analogs of nitrophenols used in plant growth biostimulants. The adsorptive potential of the studied materials reached 0.102, 0.089, 0.140, and 0.074 g of the nitrophenols g^−1^, for SiO_2_-TBD, SiO_2_-EDA, polymer-phosphazene, and PMVEAMA-PAMAM, respectively. The sorptive efficiency of the analytes, i.e., their adsorption on the functional materials, the desorption from the obtained [(sorbent)H^+^ − nitrophenolates^–^] complexes, and interactions with the used soil, were monitored using mass spectrometry (MS) technique with electrospray (ESI) and flowing atmosphere-pressure afterglow (FAPA) ionizations, for the analysis of the aqueous solutions and the solids, respectively. The results showed that the adsorption/desorption progress is determined by the structures of the terminal basic domains anchored to the materials, which are connected with the strength of the proton exchange between the sorbents and nitrophenols. Moreover, the conducted comprehensive MS analyses, performed for both solid and aqueous samples, gave a broad insight into the interactions of the biostimulants and the presented functional materials.

## 1. Introduction

The application of organic-inorganic hybrid materials or functionalized polymers containing molecular receptors capable of binding organic compounds (analytes) has gained significant attention through the last years [1,2,3,4,5]. This interest is driven mostly by the possibility of creating new materials with defined physicochemical properties with simultaneous preservation of the properties of each component, but also by the simplicity and low costs of production, stability of the final materials, and a wide application range in, e.g., analytical and applied chemistry, optics, or electronics [6,7]. The mentioned preservation of the properties of the used organic receptors is also connected with the choice of defined synthesis conditions, which should not generate any inconvenient side reactions, such as a disintegration of the organic domain or a loss of its supramolecular character providing the character of a host in the formation of host–guest complexes. Moreover, the usefulness of hybrid and/or polymeric functional materials is related to the known properties of both the chosen supporting materials and molecular receptors. Specifically, materials containing organic domains capable of the formation of non-covalent guest–host interactions may find various applications, both in the form of ‘empty’ hybrid materials with free pendant host molecules and in the form of host–guest complexes. Such application potential, at the stage of synthesis and final analytical experiments, might be investigated using various techniques; however, mass spectrometry (MS) methods are the ones of the highest reliability, sensitivity, and versatility.

One of the groups of compounds, which exhibit simultaneous agricultural application and human toxicity, are biostimulants, products of diverse formulations that positively affect the vitality of plants as a result of induced plant’s self-healing in unfavorable conditions and tolerance to stresses [8]. The most popular, widely used biostimulants are the universal Atonik biostimulator (Japan), known as Chapperone in the US, or Asahi SL, known as Superplon K in Poland. The chemical composition of the exemplary nitrophenol biostimulator contains three nitrophenolic active substances naturally found in plants: *o*-nitrophenolate, *p*-nitrophenolate, and sodium 2-methoxy-5-nitrophenolate (5-nitroguaiacolate). It has been proven to have a beneficial effect on the quality and yield of vegetable plants when they are exposed to, e.g., extremely low or high temperatures. Therefore, such commercial biostimulants have become more and more popular in recent years. The currently used agrochemical contains three phenolic compounds: sodium/potassium *p*-nitrophenolate (0.25–0.3%), *o*-nitrophenolate (0.14–0.2%), and 5-nitroguaiacolate (0.07–0.1%) [9]. Nevertheless, the positively evidenced nitrophenolic compounds, as plants’ quality-enhancing agents, are listed among the high-priority toxic pollutants presented by the U.S. Environmental Protection Agency (EPA) [10]. For instance, highly stable and water-soluble *p*-nitrophenol (*p*-NP) exhibits documented acute aquatic toxicity towards fishes, daphnias, and algae, reaching LC50 (the lethal dose required to kill 50% of members) of even 517 mg L^−1^ for a group of trout [11]. Other components of the biostimulants are sodium or potassium salts of various nitrophenols, which exhibit weak acidic character, i.e., pKa of water solutions of *p*-nitrophenol, *o*-nitrophenol, and 2-methoxy-5-nitrophenol is 7.15, 7.23, and 8.31, respectively, measured at 25 °C [12]. The use of both pure nitrophenols and their salts in plant crops is always associated with two problems: (1) how to control the dosage of biostimulator in agriculture and (2) which analytical methods should be used to control the content of nitrocompounds in waters and soils.

The controlled release of bioactive compounds from hybrid materials or functionalized polymers is studied not only in the field of biomedicine [13,14,15] but also in agriculture [16,17,18,19]. In the case of nitrophenols, there are no sufficient studies on their controlled delivery using hybrid materials as transporting platforms. However, in recent years, various analytical techniques have been used for the determination of isomers of nitrophenol, such as fluorescence detection, high-performance liquid and gas chromatography, spectrophotometry, capillary electrophoresis, and electrochemical methods, which facilitate the studies on nitrophenols’ delivery [20,21,22]. One of the first attempts of determination of phenolic compounds was presented by Geissler et al. [23], who described a method for the analysis of phenols and nitrophenols in water samples at 0.1 and 0.25 μg L^−1^ of their concentration, which involved continuous liquid-liquid extraction with a light phase rotary perforator, derivatization with either diazomethane or trimethylsulfonium hydroxide (TMSH), and analysis by GC-MS. Another example is a two-step liquid-phase microextraction (LPME) method for the extraction of phenols in environmental water samples, presented by Zhang et al. [24]. Firstly, the polar phenol (donor phase) is transferred to 1-octanol (extraction mesophase) and subsequently extracted back into a sodium hydroxide solution (acceptor phase) by vortex-assisted LPME. By the combination of the two steps of the extraction, the enrichment factors were multiplied. Under the optimized conditions, the linearities’ determination was 0.01^−1^ μg mL^−1^ for different phenols and the limits of detection were in the range from 0.3 to 3.0 ng mL^−1^ for these analytes. Furthermore, a stir bar sorptive extraction (SBSE) coupled to gas chromatography with mass spectrometry (GC-MS) using a thermal desorption unit (TDU) was implemented by Pastor-Belda et al. [25]. The limits of detection were between 0.001 and 0.031 µg L^−1^ for water and 0.020–0.107 ng g^−1^ for soil samples. When environmental samples of different origins were analyzed, contents in the 0.01–1.0 µg L^−1^ and 0.7–40 ng g^−1^ ranges were obtained for waters and soils, respectively. Additionally, a technique of spectrophotometric determination of *p*-nitrophenol was developed under the interfering impact of nano-Fe(OH)_3_ [26].

The following article presents the use of four different materials consisting of silica particles or polymer as the supports and poly(amidoamine) (PAMAM) dendrimer or strong base as the functionalizing agents. These four materials were studied for their capability of binding of the chosen nitrophenols, with a further investigation of the analytes’ release in aqueous media, using mass spectrometry measurements performed in electrospray (ESI) or flowing atmosphere-pressure afterglow (FAPA) ionization modes. Moreover, the materials were also applied for the determination of nitrophenolic compounds in various matrices.

## 2. Materials and Methods

### 2.1. Chemicals

All the reagents used for the synthesis of PAMAM-type dendrimers, the strong bases used for the assessment of their interactions with nitrophenols (TBD (1,5,7-triazabicyclo[4.4.0]dec-5-ene) and phosphazene (2-*tert*-Butylimino-2-diethylamino-1,3-dimethylperhydro-1,3,2-diazaphosphorine)), poly(methyl vinyl ether-*alt*-maleic anhydride) (PMVEAMA) chains as a polymeric support (average MW ~216,000 Da, average Mn ~80,000 Da), and functional sorbent hereinafter called polymer-phosphazene (Table 1) (2-*tert*-Butylimino-2-diethylamino-1,3-dimethylperhydro-1,3,2-diazaphospho-rine, polymer-bound; 200–400 mesh, extent of labeling: 2.0–2.5 mmol g^−1^ loading, 1% cross-linked) were obtained from Sigma Aldrich (St. Louis, MO, USA). Silica particles functionalized with maleimide residues as a linker for silica-based PAMAM-grafted material (particles’ size: 40–63 μm, loading: 0.68 mmol g^−1^) and silica particles containing surface reactive TBD groups, treated as a sorbent (Table 1) (particles’ size: 40–63 μm, loading: 0.89 mmol g^−1^), were purchased from SiliCycle Inc. (Québec, QC, Canada). All the solvents were of purity grade p.a. and used with no purification.

### 2.2. The Synthesis of the Molecular Receptors and the Functional Materials

#### 2.2.1. The Synthesis of PAMAM-Type Dendrimers

Two kinds of PAMAM dendrimers containing tris(2-aminoethyl)amine as an amine core and ethylenediamine as a terminal amine component (EDA; MW = 831 Da) and diethylenetriamine as both core and terminal amine component (DETA; MW = 888 Da) were obtained as described in our previous articles [27,28]. The synthetic strategy was based on the two-step approach, involving: (1) the branching of a core “initiator” molecule, which contained functional amine groups, with methyl acrylate (branching unit) following Michael addition, and (2) outward growth of the dendrimers accomplished by amidation of an ester-intermediate with a particular diamine.

#### 2.2.2. The Synthesis of Dendrimer-Functionalized Materials

The silica-based material functionalized with EDA dendrimer was obtained and characterized according to the procedure described in our previous article [27]. Briefly, EDA dendrimer (0.52 g; 0.8 mmol) was dissolved in 40 mL of DMF. The solution was purged with nitrogen, and then heated to approximately 75 °C. Then, 2 g of maleimide-modified silica particles were added to the heated solution in a few portions, and the mixing in an inert atmosphere of nitrogen at elevated temperature was maintained for 5 h. Afterward, the warm mixture was filtered off, and the solid was washed with DMF (30 mL) and DCM (25 mL), obtaining a yellow-orange material (Table 1).

Moreover, PMVEAMA-DETA polymeric material was obtained and characterized according to the procedure described in [28]. Briefly, a solution of DETA dendrimer (4.55 g; 5.12 mmol) dissolved in 40 mL of DMSO was placed in a three-necked flask equipped with a reflux condenser. The solution was purged with nitrogen and heated to 110 °C. Then, a suspension of PMVEAMA (1.6 g) in 30 mL of toluene was added to the heated solution of the dendrimer in a few portions. The stirring was continued for 16 h under the atmosphere of nitrogen. Subsequently, the solvents were evaporated under reduced pressure and the crude product was precipitated using diethyl ether. The resulting green-blue material was filtered off, washed several times with cold diethyl ether, and dried under vacuum at 50 °C. The structure of the polymeric functional material is presented in Table 1.

### 2.3. Instruments

The ESI-MS spectra were recorded using an amaZon SL ion trap (Bruker, Bremen, Germany) equipped with an electrospray ion source in infusion mode. The sample solution was introduced into the ionization source at a flow rate of 5 μL min^−1^ using a syringe pump. The apparatus was operated using the so-called “enhanced resolution mode” (mass range: 50–2200 m/z, scanning rate: 8100 m/z per second). The capillary voltage was set at +4.5 kV and the endplate offset at −500 V. The source temperature was 80 °C and the desolvation temperature was 250 °C. Nitrogen was used as the cone gas and desolvating gas (helium) at flow rates of 800 L h^−1^ and 50 L h^−1^, respectively. The mass spectrometer was operated in the ESI positive and negative ionization mode. Mass spectrometers were equipped optionally in V-FAPA ambient plasma source (ERTEC, Wroclaw, Poland). The application of the flowing atmospheric-pressure afterglow (FAPA) ion source enabled the high-energy helium atom He (2^3^S) to be used for ions’ generation. Oxygen, nitrogen, water, and analytes, in contact with the high-energy helium atom He (2^3^S), played key roles in chemical and physical formation of ions in FAPA ionization. The production of N_2_^+^, O_2_^+^, NO^+^, and ions of analytes in this process resulted from the interactions between air and water molecules with He (2^3^S) [29]. In negative ion mode, ions were produced by the interaction of thermal electrons in the afterglow region with ambient oxygen. Gas-phase compounds with a higher gas-phase acidity than O_2_^−^ were deprotonated, producing [M−H]^−^ ions [30].

**Figure 1:**
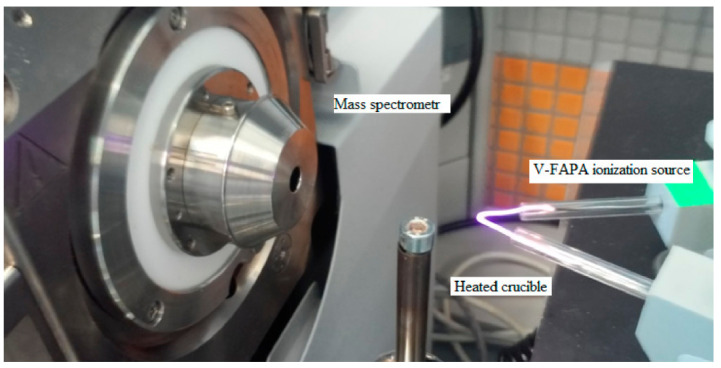
The measuring system consisting of the V-FAPA ionization source (**top right**), the heated crucible (**bottom right**), and the mass spectrometer (**left**).

The flowing atmospheric-pressure afterglow (FAPA) ion source for *mass* spectrometry was used to generate plasma, allowing the ionization of analyte particles released thermally from a heated crucible with temperature regulation from 20 to 400 °C, at a temperature increase rate of 3 °C s^−1^ (Figure 1). The temperature of the maximum desorption of the analyte was 340 ± 10 °C. The mini crucible allowing the temperature-controlled desorption was placed ca. 10 mm below the ion stream. The distance between the inlet to the mass spectrometer and the FAPA ion source was about 10 cm [31,32].

### 2.4. The Sorption of Nitrophenols-Containing Biostimulator Using the Tested Functional Materials

#### 2.4.1. Adsorption Experiments

The adsorption experiments involved the use of a stock solution of the biostimulator in deionized water containing *p*-nitrophenol and 2-methoxy-5-nitrophenol in the mass ratio of 1:1, with the concentrations of each nitrophenol of 1.4 × 10^−1^ g L^−1^. To a series of 10 mL aqueous mixtures of the nitrophenols, concentrations of which ranged between 1.4 × 10^−8^ g L^−1^ to 1.4 × 10^−1^ g L^−1^, 100-mg samples of sorbent (SiO_2_-TBD, SiO_2_-EDA, Polymer-Phosphazene, or PMVEAMA-DETA) were added and the mixtures were stirred with a magnetic stirrer for 5 min at room temperature. Afterward, the materials were centrifuged, and the resulted solutes and solids were analyzed using ESI-MS and FAPA-MS analysis, respectively. The analytical signals of the nitrophenols on the MS spectra were m/z 138 for *p*-nitrophenol and m/z 168 for 2-methoxy-5-nitrophenol, both present in negative mode. The performed ESI-MS allowed for the calculation of the amount of the nitrophenols remaining in the solution after the adsorption process, on the basis of pre-performed calibration curves, while FAPA-MS analysis of 5-mg samples allowed for the quantification of the amount of the nitrophenols adsorbed on the materials.

#### 2.4.2. The Release of the Nitrophenols from the Functional Sorbents

The obtained complexes of the materials and surface-adsorbed nitrophenols were further subjected to the release of the biostimulator’s ingredients. Therefore, 100-mg samples of sorbent-nitrophenols were placed in 25-mL glass vials, and 10 mL of the releasing medium, deionized water (pH = 6.7), was added. During the mixing of the samples at room temperature using a magnetic stirrer, 20-μL aliquots of the solute were collected after 1, 24, and 48 h of incubation, and analyzed for the amount of the nitrophenols released, using ESI-MS analysis. Additionally, after 48 h of incubation, the materials were centrifuged, and then 5-mg samples were subjected to direct FAPA-MS analysis, in order to calculate the amount of the nitrophenols remaining in the sorbents [33,34].

#### 2.4.3. Analysis of the Nitrophenols’ Affinity towards Soil

The affinity of the biostimulator’s components towards the soil was assessed by mixing of 100 g of the soil with a series of three 10-mL solutions of mixtures of the biostimulator, which contained the biostimulating ingredients at the concentrations of 0.5 × 10^−6^, 1 × 10^−6^, and 1.5 × 10^−6^ g per g of the soil, which was 0.36, 0.71, and 1.07 mL of the biostimulator solution of 1.4 × 10^−1^ g L^−1^, respectively. The soil–biostimulator mixtures were mixed for 24 h at room temperature and then placed in a funnel. The separated enriched soil was then washed on the funnel with 500 mL of distilled water, leading to the separated soil and the soil extract containing washed nitrophenols. The dried soil sample of 5 mg was analyzed with the FAPA-MS technique for the determination of the remaining nitrophenols’ content in the soil. Moreover, a series of four 10-mL samples of the soil extract were incubated for 30 min at room temperature with 100-mg samples of the studied sorbents. The content of bound and remaining biostimulator ingredients was analyzed using the FAPA-MS method.

## 3. Results

The introduction of particular organic receptors on the surface of chosen supports, as binding domains, must be initially justified by their ability to interact with the analytes, which are intended to be bound by the final adsorbents. For this purpose, interactions between strong bases used as functionalizing agents of the studied sorbents (TBD, phosphazene, and PAMAM-type dendrimers) and nitrophenolates, as the chosen analytes, were studied using ESI-MS measurements. The studies involved the analysis of mixtures of an aqueous solution containing a single, strong base used and the mixture of nitrophenols. The positive ESI-MS spectra presented monopositive signals at m/z 140, 275, 832, and 889, which corresponded to protonated forms [TBD + H]^+^, [phosphazene + H]^+^, [EDA + H]^+^, and [DETA + H]^+^, respectively, while, in the range of negative ions, the observable signals were at m/z 138 related to [*p*-nitrophenolate]^−^ and m/z 168, corresponding to ([2-methoxy-5-nitrophenolate]^−^) forms. Therefore, the strong bases and the studied nitrophenols interacted by proton exchange, leading to ionic pairs (complexes):

Base + Nitrophenol ⇌ Base−H^+^ + Nitrophenolate^−^

**Figure 2:**
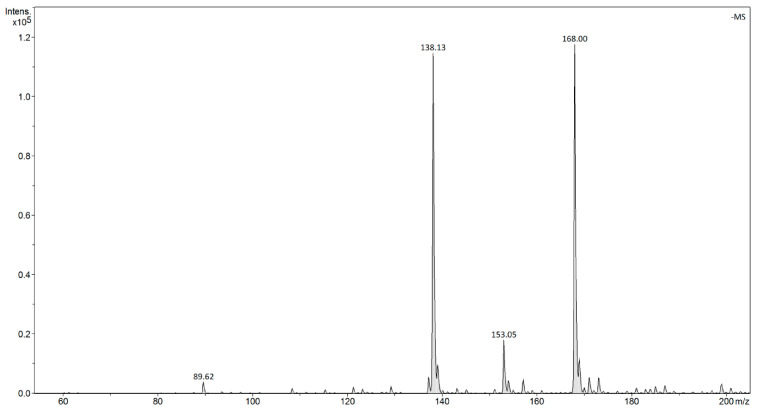
The ESI-MS spectrum of the aqueous mixture of the nitrophenols at their concentrations of 1.4 × 10^−7^ g L^−1^; m/z 138.13 [*p*-nitrophenol-H]^−^, 168.00 [2-methoxy-5-nitrophenol-H]^−^.

**Figure 3:**
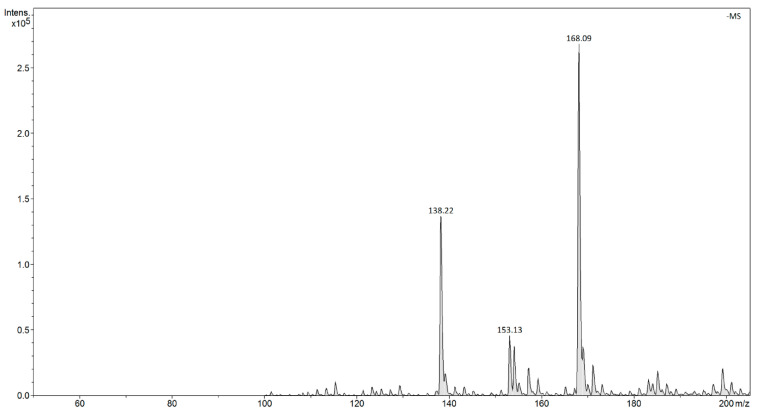
The FAPA-MS spectrum of the aqueous mixture of the nitrophenols at their concentrations of 1.4 × 10^−7^ g L^−1^; m/z 138.22 [*p*-nitrophenol-H]^−^, 168.09 [2-methoxy-5-nitrophenol-H]^−^.

An investigation of dissociated Base-H^+^-Nitrophenolate^−^ products in water by ESI mass spectrometry showed that no negative influence of a strong base on the determination and intensity of signals m/z of the nitrophenolate anion were observed. Therefore, two inorganic-organic hybrid materials (SiO_2_-TBD and SiO_2_-EDA) and two polymeric materials (Polymer-Phosphazene and PMVEAMA-PAMAM) containing pendant, either a strong base residue or a dendritic structure as receptors capable of binding nitrophenols, were tested. In order to assess the adsorptive properties of the tested hybrid and polymeric materials towards nitrophenols, two different techniques of mass spectrometry were implemented. The ESI mass spectrometry enabled direct tracking of changes in the concentration of the nitrophenols in the solutions after binding of the analytes within the matrices of hybrid and polymeric sorbents and preceded the separation (most usually centrifugation) of the material. On the other hand, FAPA mass spectrometry allowed both the monitoring of the concentration changes in the solutions (analogous to ESI mode) and also qualitative and quantitative analysis of the analytes bound to a solid sorbent by its thermal desorption from the material. The use of both techniques in an analytical procedure gives a deep insight into the evaluation of adsorptive processes between the nitrophenols and the functional materials. In both ESI- and FAPA-MS analyses, the negative ion signals at m/z 138 and 169 were selected as the analytical signals for *p*-nitrophenol and 2-methoxy-5-nitrophenol, respectively, which corresponded to the deprotonated structure of the nitrophenols. The intensities of these signals were, therefore, used for the quantitative analysis of the adsorption of the nitrophenols, by the preparation of calibration curves allowing for the monitoring of changes in the analytes’ concentration in aqueous samples. Exemplary spectra of ESI- and FAPA-MS are shown in Figure 2 and Figure 3, respectively.

**Figure 4:**
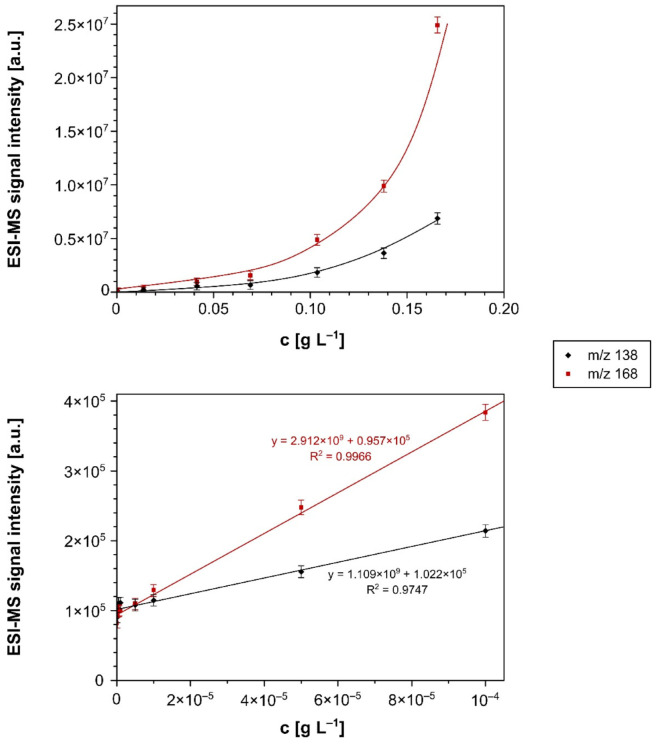
The ESI-MS calibration curves for *p*-nitrophenol (m/z 138) and 2-methoxy-5-nitrophenol (m/z 168), performed in the two concentration ranges: 1 × 10^−8^ to 2 × 10^−1^ g L^−1^ (**top**) and 1 × 10^−8^ to1 × 10^−4^ g L^−1^ (**bottom**).

**Figure 5:**
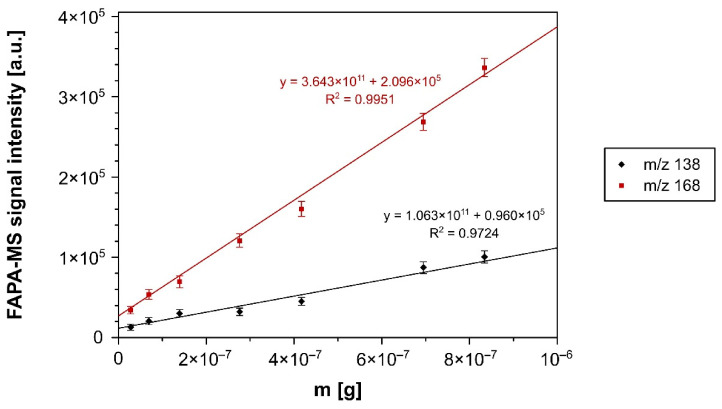
The FAPA-MS calibration curves for *p*-nitrophenol (m/z 138) and 2-methoxy-5-nitrophenol (m/z 168), performed in the mass range: 2 × 10^−8^ to 9 × 10^−7^ g.

For the quantitative analysis, the initial stage of the studies involved the preparation of calibration curves presented as the dependence of m/z 138 and 168 [M − H]^−^ signal intensities recorded in the negative ions range vs. the concentration of the corresponding nitrophenol. The calibration curves were obtained using the recorded MS spectra performed in both ESI and FAPA ionization techniques, which are presented in Figure 4 and Figure 5, respectively. Each measurement was carried out three times. On their basis, the mean values of the signal intensities and their standard deviations (not exceeding 10% of the intensity values) were calculated.

On the basis of the obtained curves, the linearity ranges of the methods in the negative mode were set at 2 × 10^−8^ to 1 × 10^−7^ g L^−1^ for ESI-MS measurements and at 2 × 10^−8^ to 1 × 10^−7^ g L^−1^ for FAPA-MS technique. The use of new measurement techniques and the use of new materials required the determination of the limit of detection (LOD) experimentally, which is usually defined as the lowest amount or concentration of a component that can be reliably detected by a given analytical method. In order to determine the LOD value, we used the method described in the previous publication [35]. Each analytical instrument produces a signal even when a mixture containing no component (blank) is analyzed. To determine the LODs, we measured the signal intensities corresponding to the nitrophenols’ signals, with no nitrophenols in the samples (referred to as the noise floor), five times in a row. Then the standard deviation (SD) of these measurements was calculated. According to the LOD definition (LOD = mean blank value + 3·SD), if the mean signal intensity at m/z 138 and 168 exceeds this value, the analyte is in the detection range. The limits of detection of both nitrophenols were 2 × 10^−7^ and 5 × 10^−9^ g L^−1^ for ESI-MS and FAPA-MS, respectively.

In the next step of the studies, the chosen four materials containing pendant strong base residues (SiO_2_-TBD, SiO_2_-EDA, Polymer-Phosphazene, and PMVEAMA-DETA) were investigated for the adsorption of the nitrophenols from their aqueous solutions. The concentrations of the nitrophenols remaining in the solutions after the adsorption processes were determined using the ESI-MS method, which are collected in Table 2, while the amount of the nitrophenols adsorbed to the materials’ structure was determined using direct FAPA-MS analysis of the material-nitrophenols solids (Table 3). The use of these two analytical methods allowed for the investigation of the adsorption progress monitored in both solutes and solids. Moreover, the used FAPA-MS technique enabled fast and direct determination of the components adsorbed on the sorbent, with no analytes’ release steps. The amount of the adsorbed *p*-nitrophenol and 2-methoxy-5-nitro- phenol on the tested sorbents corresponded to the composition of the solutions used to obtain the (adsorbent-H^+^ + nitrophenolate^–^) complexes, and were within the error limits.

As is presented in Table 2 and Table 3, the amount of the nitrophenols adsorbed to the sorptive materials depended primarily on the quality of interactions between the nitrophenols and the organic receptors deposited on the hybrid and polymeric functional materials. Therefore, the stronger the base anchored on the sorbent’s surface, the higher the amount of bound biostimulating agents. Moreover, the presented method needed to be tested for its reproducibility/repeatability, which might be defined as a degree of the compliance of successive measurement results of the same measurements, performed under the same conditions. In order to determine the repeatability, the determination of nitrophenols in water was carried out for four times using the same mass of adsorbents and two different concentrations of the nitrophenols. The corresponding results presented in Table 4 indicate a good reproducibility of the implemented method.

**Figure 6:**
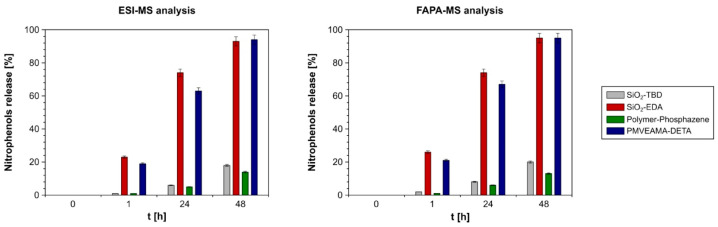
The release of the nitrophenols from [adsorbent-H^+^ + nitrophenolate^−^] complexes, investigated using two different MS analyses: ESI-MS (**left**) and FAPA-MS (**right**).

The obtained complexes (adsorbent-H^+^ + nitrophenolate^–^) may also release the studied nitrophenols from their structure, under certain conditions, into water or soil and play a role of biostimulators of cultivated plants. Therefore, the complexes of the functional materials after their incubation in the most concentrated solution of the nitrophenols (initial concentration of each nitrophenol of 1.4 × 10^−1^ g L^−1^) were subjected to release experiments. The calculated mean amount of the nitrophenols bound to the materials were 0.102, 0.089, 0.140, and 0.074 g of each nitrophenol g^−1^ material, for SiO_2_-TBD, SiO_2_-EDA, Polymer-Phosphazene, and PMVEAMA-DETA, respectively. It is worthy to highlight that the differences between the amount of *p*-nitrophenol and 2-methoxy-5-nitrophenol were rather insignificant, which was a result of the similar pK_a_ values of these compounds, not increasing the selectiveness of their interactions with the pendant basic receptors. The nitrophenols’ release progress was monitored by two complementary methods of mass spectrometry, i.e., the ESI-MS measurements of the water solutions during the incubation of the complexes in deionized water in preset time intervals (1, 24, and 48 h) and FAPA-MS analysis of the isolated materials from deionized water in the same time intervals. The dependence of the release percentage as a function of time is presented in Figure 6. 

The cumulative nitrophenols’ release to deionized water samples, studied using ESI-MS analysis after 48-h incubation, was 20%, 95%, 13%, and 95% for the complexes of SiO_2_-TBD-H^+^-, SiO_2_-EDA-H^+^-, Polymer-Phosphazene-H^+^-, and PMVEAMA-DETA-H^+^- nitrophenolate^−^, respectively. The corresponding values, calculated for the measurements involving the FAPA-MS method, were 17%, 98%, 9%, and 94%, which showed the reliability of both analytical procedures. However, both sets of values obtained using different mass spectrometric techniques confirmed that the percentage of nitrophenols’ release depends on the strength of interactions between basic residue on the surface of the materials and the nitrophenols. The presence of the strongest bases (TBD and phosphazene) on the functional materials’ surface led to the most intensive binding of the analytes in their structure, but also to the least effective release of the nitrophenols to the releasing medium, which reached only 19% and 14%, respectively, after 48-h incubation.

Furthermore, the quantitative potential of the studied materials triggered their implementation as analytical tools for the analysis of the nitrophenols after a soil enrichment. The use of nitrophenols as growth biostimulants in agriculture is becoming a more and more common treatment. Manufacturers of this type of preparation highlight the dependence of dosage on a type of plant cultivation; however, they recommend an optimal concentration of the commercial biostimulants of 1.5 × 10^−6^ g per g of soil. Such concentration is widely used during the spraying of plants in order to obtain the effects of biostimulation. For this reason, the studied materials were implemented for the determination of the nitrophenols’ content in the soil extract of an enriched soil with a biostimulator solution. The recovery of the nitrophenols from soil extracts, which were derived from the soil enriched with the biostimulants at three different concentrations, 0.5 × 10^−6^, 1 × 10^−6^, and 1.5 × 10^−6^ g of nitrophenols per g of the soil, was monitored using FAPA-MS method. The calculated recovery values were 42%, 47%, 34%, and 44% for the used SiO_2_-TBD, SiO_2_-EDA, Polymer-Phosphazene, and PMVEAMA-DETA sorbents, respectively. The low values might result from strong sorptive properties of the soil towards the studied nitrophenols, which indicates the ineffectiveness of washing out the nitrophenols from the soil with water. Moreover, the separated sorptive materials, after the treatment with the soil extract, were found to bind a number of other acid species contained in the soil. This result showed that the analysis of the nitrophenols in the soil and its extract using the materials containing basic organic domains was rather unsatisfactory, and new analytical methods must be implemented.

## 4. Conclusions

In this article, we presented two functional silica-based materials and two polymeric materials, which contained structurally different strong base residues deposited on their surface, which were PAMAM dendrimers, TBD, and phosphazene. The strongly basic character of the pendant organic receptors triggered the implementation of the studied materials as sorbents dedicated to nitrophenols (*p*-nitrophenol and 2-methoxy-5-nitrophenol), which exhibit applicability in agriculture as plant growth biostimulants. The adsorption of the nitrophenols on the studied functional materials was monitored using MS measurements performed in two ionization techniques: ESI and FAPA. The main advantage of such an analytical approach is the ability to comprehensively investigate the changes in the analytes’ concentration in both the studied solutes and solid phase. The materials showed different sorption capacities, ranging between 0.074 and 0.140 g g^−1^, which were dependent on the basicity of the introduced surface receptors since the adsorption was driven by a proton exchange between them and the used nitrophenols. Moreover, the materials were also studied as analytical tools dedicated to the analysis of soil extracts, derived from the biostimulants-treated agriculture soil. However, due to the low extraction efficiency of nitrophenols and the ability of sorbents to interact with other acid compounds, the proposed method is limited to qualitative analysis.

## Figures and Tables

**Table 1:** The structures of the studied sorbents.

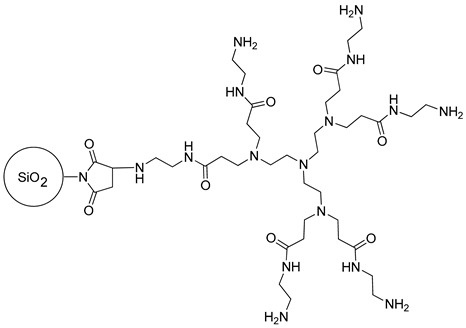	
SiO_2_-EDA	
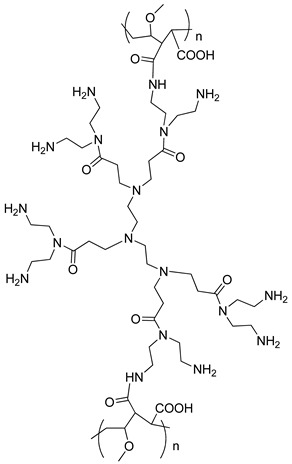	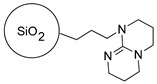
	SiO_2_-TBD
	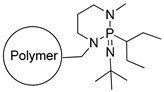
PMVEAMA-DETA	Polymer-Phosphazene

**Table 2:** The concentrations of *p*-nitrophenol (*p*-NP) and 2-methoxy-5-nitrophenol (MNP) remaining in the solution after the adsorption processes, monitored using ESI-MS analysis (*c*_0_ relates to the initial concentrations of each nitrophenol).

	Concentration of Nitrophenols in Solution after Adsorption [g L^−1^]							
	SiO_2_-TBD		SiO_2_-EDA		Polymer-Phosphazene		PMVEAMA-DETA	
*c*_0_ [g L**^−^**^1^]	*p*-NP	MNP	*p*-NP	MNP	*p*-NP	MNP	*p*-NP	MNP
1.4 × 10**^−^**^1^	4.3 × 10^−^^3^	3.9 × 10^−^^3^	5.3 × 10^−^^3^	5.9 × 10^−^^3^	1.2 × 10^−^^3^	1.1 × 10^−^^3^	5.8 × 10^−^^3^	5.9 × 10^−^^3^
1.4 × 10**^−^**^2^	4.6 × 10^−^^4^	4.1 × 10^−^^4^	6.6 × 10^−^^4^	6.3 × 10^−^^4^	2.6 × 10^−^^4^	2.4 × 10^−^^4^	7.0 × 10^−^^4^	7.0 × 10^−^^4^
1.4 × 10**^−^**^3^	1.2 × 10^−^^5^	1.4 × 10^−^^5^	4.2 × 10^−^^5^	4.1 × 10^−^^5^	0.9 × 10^−^^5^	1.1 × 10^−^^5^	5.1 × 10^−^^5^	4.9 × 10^−^^5^
1.4 × 10^−^^4^	1.1 × 10^−^^6^	0.9 × 10^−^^6^	2.2 × 10^−^^6^	1.9 × 10^−^^6^	0.9 × 10^−6^	0.6 × 10^−^^6^	2.8 × 10^−^^6^	2.9 × 10^−^^6^
1.4 × 10^−^^5^	2.1 × 10^−^^7^	2.0 × 10^−^^7^	4.1 × 10^−^^7^	4.2 × 10^−^^7^	nd	nd	4.9 × 10^−^^7^	4.9 × 10^−^^7^

nd—non detected.

**Table 3:** The amount of *p*-nitrophenol (*p*-NP) and 2-methoxy-5-nitrophenol (MNP) in the materials after the adsorption processes, monitored using FAPA-MS analysis (*c_0_* relates to the initial concentrations of each nitrophenol, which are: (a) 1.4 × 10^−1^; (b) 1.4 × 10^−3^; and (c) 1.4 × 10^−5^ g L^−1^).

	The Amount of the Nitrophenols Adsorbed on the Functional Materials [g g^−1^] with Indicated Adsorption Percentages							
	SiO_2_-TBD		SiO_2_-EDA		Polymer-Phosphazene		PMVEAMA-DETA	
	*p*-NP	MNP	*p*-NP	MNP	*p*-NP	MNP	*p*-NP	MNP
(a)	1.02 × 10^−1^	1.01 × 10^−1^	8.94 × 10^−2^	8.84 × 10^−2^	1.40 × 10^−1^	1.41 × 10^−1^	7.24 × 10^−2^	7.61 × 10^−2^
	(72.8%)	(72.1%)	(63.8%)	(63.1%)	(100.0%)	(100.7%)	(53.00%)	(54.4%)
(b)	1.28 × 10^−3^	1.34 × 10^−3^	9.97 × 10^−4^	9.23 × 10^−4^	1.39 × 10^−3^	1.36 × 10^−3^	8.42 × 10^−4^	8.91 × 10^−3^
	(91.4%)	(95.7%)	(71.2%)	(65.9%)	(99.2%)	(97.1%)	(60.1%)	(63.6%)
(c)	1.42 × 10^−5^	1.54 × 10^−5^	8.90 × 10^−5^	8.57 × 10^−5^	1.42 × 10^−5^	1.46 × 10^−5^	7.44 × 10^−5^	7.64 × 10^−5^
	(101%)	(110%)	(63.5%)	(40.7%)	(101.4%)	(104.3%)	(53.1%)	(54.6%)

**Table 4:** The reproducibility tests showing the calculated concentrations of the nitrophenols adsorbed from their 1 × 10^−6^ and 1.4 × 10^−5^ g L^−1^ solutions by the sorbents, using FAPA-MS measurements.

Concentration of Nitrophenols in Water Solution [g L^−1^]		The Calculated Concentrations of the Nitrophenols in Water Solutions with the Use of Adsorbents, Detected by the FAPA MS Technique			
		SiO_2_-TBD	SiO_2_-EDA	Polymer-Phosphazene	PMVEAMA-DETA
p-NP MNP	1 × 10^−6^ 1 × 10^−6^	(0.92–1.10) × 10^−6^ (0.91–1.14) × 10^−6^	(0.89–1.12) × 10^−6^ (0.92–1.13) × 10^−6^	(0.90–1.15) × 10^−6^ (0.88–1.14) × 10^−6^	(0.97–1.12) × 10^−6^ (0.90–1.16) × 10^−6^
p-NP MNP	1.4 × 10^−5^ 1.4 × 10^−5^	(1.38–1.54) × 10^−5^ (1.28–1.58) × 10^−5^	(1.32–1.54) × 10^−5^ (1.21–1.59) × 10^−5^	(1.37–1.53) × 10^−5^ (1.31–1.58) × 10^−5^	(1.31–1.56) × 10^−5^ (1.28–1.56) × 10^−5^

## Data Availability

The data presented in this study are available on request from the corresponding author.
